# Central nervous system histopathological findings in classic infantile Pompe disease: a systematic review with clinical relevance

**DOI:** 10.1007/s00415-026-13991-y

**Published:** 2026-07-31

**Authors:** Jan J. A. van den Dorpel, Martha C. Faraguna, Robert M. Verdijk, Nadine A. M. E. van der Beek, W. W. M. Pim Pijnappel, Ans T. van der Ploeg, Johanna M. P. van den Hout

**Affiliations:** 1 Center for Lysosomal and Metabolic Diseases, Department of Pediatrics, Erasmus MC, University Medical Center Rotterdam, P.O. Box 2060, 3000 CB Rotterdam, The Netherlands; 2 School of Medicine and Surgery, University of Milano-Bicocca, Monza, Italy; 3 Department of Pathology, Section Neuropathology, Erasmus MC, University Medical Center Rotterdam, Rotterdam, The Netherlands; 4 Department of Pathology, Section Neuropathology, Leiden University Medical Center, Leiden, The Netherlands; 5 Center for Lysosomal and Metabolic Diseases, Department of Neurology, Erasmus MC, University Medical Center Rotterdam, Rotterdam, The Netherlands; 6 Department of Clinical Genetics, Erasmus MC, University Medical Center Rotterdam, Rotterdam, The Netherlands

**Keywords:** Classic infantile Pompe disease, Central nervous system, Brain glycogen, Astrocytes, White matter abnormalities, Gray matter disease, Long-term survivors

## Abstract

**Introduction:**

Classic infantile Pompe disease is the most severe phenotype within the clinical spectrum of Pompe disease and is characterized by central nervous system (CNS) involvement.

**Methods:**

A systematic review on histopathological findings in classic infantile Pompe disease was conducted using Embase, Medline Ovid, Web of science and Cochrane central databases for original research articles until 16-12-2025. Articles reporting a histological examination of the CNS were selected. Classic infantile Pompe disease was defined by hypertrophic cardiomyopathy, symptom onset < 12 months of age, and GAA deficiency, if available.

**Results:**

Of the 3734 records identified, 39 articles were included in the study, comprising 49 patients, 7 of whom treated with enzyme replacement therapy. The median age at autopsy was 6 months (range 1.2–17.0) for untreated and 12 months (range 7.5–21.0) for treated patients. Histologic abnormalities were found throughout the CNS. The cerebral and cerebellar white matter (WM), globus pallidus, dentate nucleus, motor nuclei of the brainstem, and anterior horn cells were most frequently affected. Glycogen accumulation was severe in astrocytes and in neurons of the globus pallidus. Cortical neurons were affected with greater variability, and subcortical structures were more frequently involved than cortical ones.

**Conclusions:**

Understanding of CNS involvement in classic infantile Pompe disease is rapidly evolving. Histopathological studies from children up to 21 months demonstrate early and widespread CNS pathology. Notably, there is a marked temporal gap between the extensive abnormalities observed at autopsy in infancy and later radiological, biochemical, and clinical manifestations. While white matter pathology is already prominent in young patients, abnormalities on magnetic resonance imaging typically emerge from 2 to 3 years of age, biomarker changes from around 5 years, and cognitive decline, reduced processing speed, and background activity slowing on electroencephalogram from approximately 8–10 years onward. These observations suggest a gradual evolution of CNS disease, although direct clinicopathological correlations remain limited. Epilepsy has been reported later in the disease course, and we have observed upper motor neuron signs in older patients, suggesting progressive gray and white matter involvement that may contribute to cognitive decline. The mechanisms linking glycogen accumulation to neurological manifestations remain incompletely understood. Taken together, these observations form the basis for several hypotheses on the pathogenesis of CNS involvement in classic infantile Pompe disease.

**Graphical abstract:**

**Figure:**
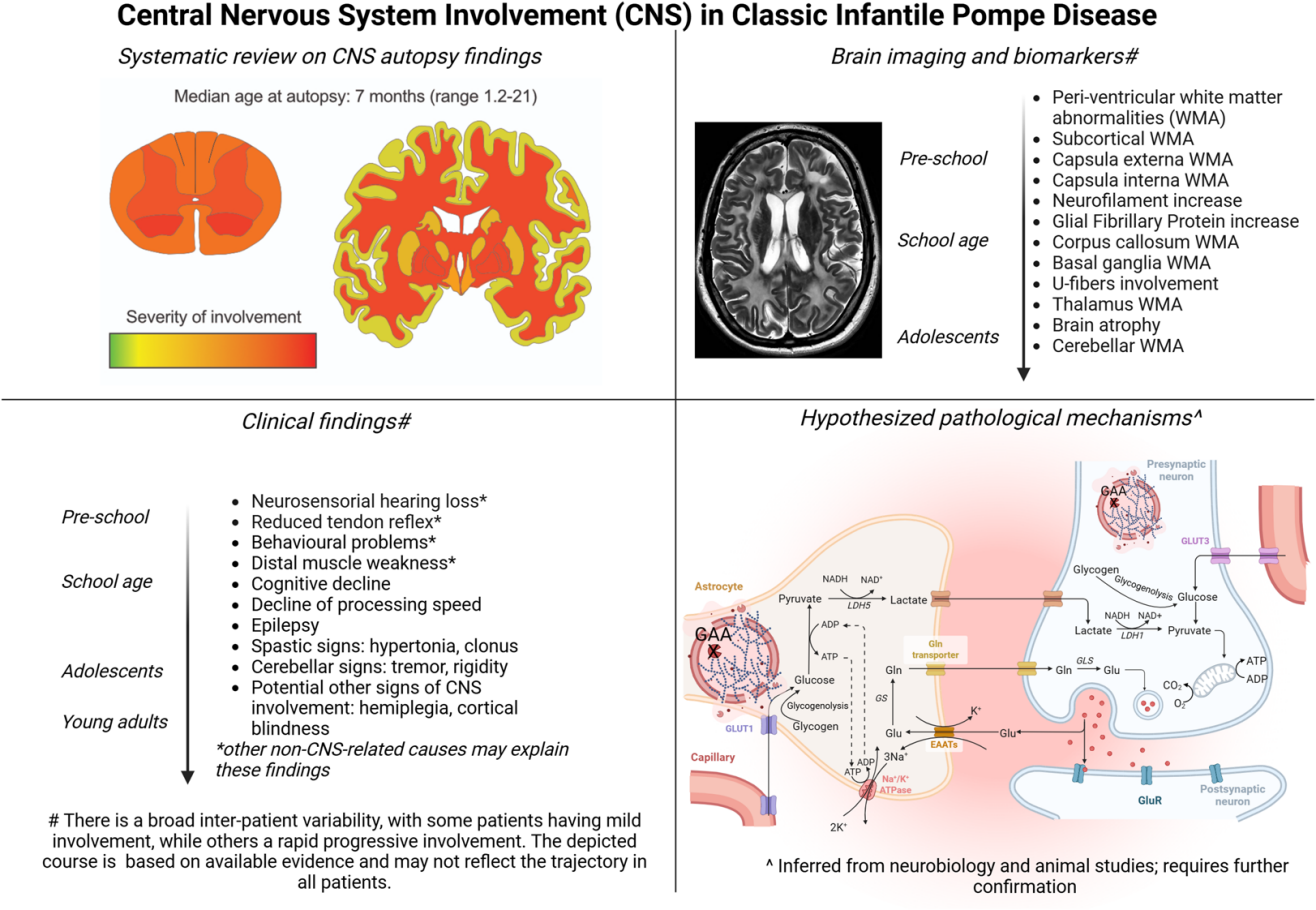

**Supplementary Information:**

The online version contains supplementary material available at 10.1007/s00415-026-13991-y.

## Introduction

Classic infantile Pompe disease is a severe, progressive metabolic disorder, in which alpha-glucosidase deficiency leads to a multisystemic glycogen accumulation, most predominantly in cardiac and skeletal muscle. It represents the most severe end of the Pompe disease phenotypical spectrum and is characterized by hypertrophic cardiomyopathy, severe generalized hypotonia, and respiratory distress [1]. The disease course of classic infantile Pompe disease, associated with premature death in the first year of life, has been greatly modified by the advent of enzyme replacement therapy (ERT) with recombinant human alglucosidase alpha. This made Pompe disease the first treatable genetic neuromuscular disorder. ERT has allowed many patients to achieve walking and some patients to reach early adulthood. However, ERT is not curative, and patients still suffer from substantial unmet medical needs, such as residual proximal and distal muscle weakness, bulbar muscle weakness, and central nervous system involvement, ranging from mild cognitive impairment to extensive white matter abnormalities, cerebral atrophy, and epilepsy [2–6]. Recombinant alglucosidase alpha and the second generation ERTs, avalglucosidase alpha and cipaglucosidase alpha, do not cross the blood–brain barrier (BBB), highlighting the urge of innovative treatments which may address the CNS involvement.

While working toward new therapies which may cross the BBB [7, 8], it is crucial to fully understand the histopathological consequences of classic infantile Pompe disease in order to identify the involved anatomical regions and cell types. Anatomical pathology reports can contribute to the elucidation of the pathophysiological sequence of events leading to CNS involvement. This literature review aims to provide a histopathological overview of the central nervous system involvement in classic infantile Pompe patients and correlate histological findings to neuroimaging studies and known clinical symptoms.

## Methods

### Search strategy

**Fig. 1:**
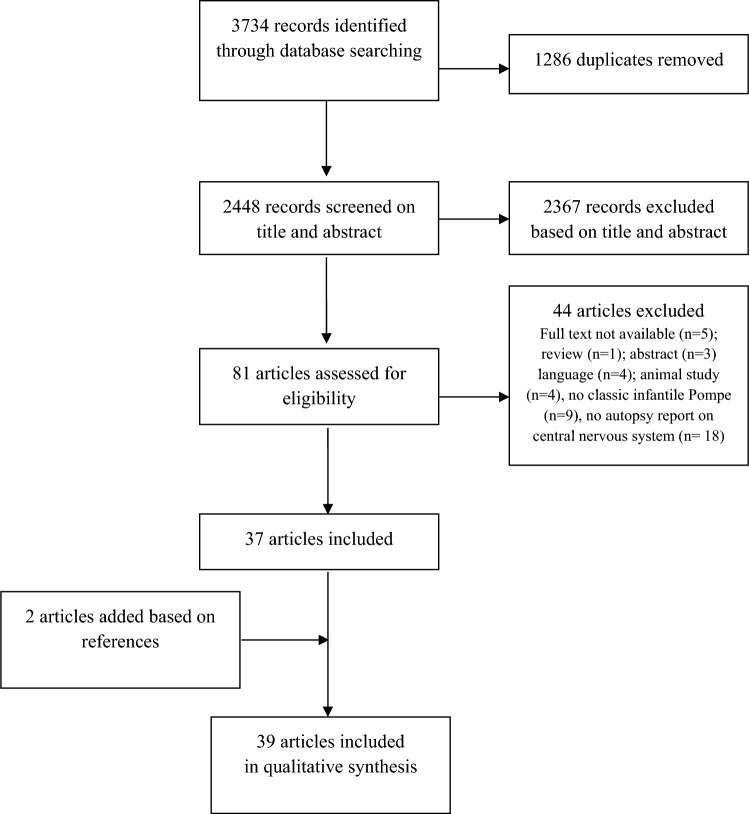
PRISMA-style flow diagram of study identification, screening, eligibility, and inclusion for the systematic review

This systematic review was conducted in accordance with the Preferred Reporting Items for Systematic Reviews and Meta-Analyses (PRISMA) guidelines, as illustrated in Fig. 1. Although no protocol was prospectively registered, the review methodology was predefined and consistently applied throughout the study. The databases Embase, Medline Ovid, Web of science and Cochrane central databases were systematically searched for the terms “Pompe disease”, “acid alpha-glucosidase deficiency”, “acid maltase deficiency” and “glycogen storage disease type II”. We conducted a separate, broader search for “glycogen storage disease” in old Embase (< 1975) and old Medline (< 1966) databases, to avoid missing articles due to incorrect indexation. All searched terms are summarized in Appendix A. All databases were searched for results from inception up to 16th December 2025.

### Selection strategy

Title and abstract screening were conducted independently by two reviewers (JvdD and JMPvdH) to identify potentially eligible studies, followed by full-text screening by both reviewers. Subsequently, data extraction was carried out independently by JD and verified by JMPvdH. Any discrepancies arising during the screening or data extraction phases were resolved through discussion and consensus.

Studies were considered eligible if they reported histological examination of the central nervous system in patients with a confirmed diagnosis of classic infantile Pompe disease. This diagnosis was defined by deficient acid alpha-glucosidase activity and/or symptom onset before one year of age in combination with hypertrophic cardiomyopathy. Studies were excluded if: (1) they were not published in English, French, German, or Dutch; (2) they were review articles or conference abstracts; (3) the full text was unavailable; or (4) the diagnosis or clinical criteria were insufficiently defined or uncertain.

In addition, the reference lists of all included studies were manually screened to identify any further relevant publications.

### Data extraction and semi-quantitative scoring system

From included studies we gathered the following clinical information: sex, age at first symptoms, age at autopsy, and presence of cardiomegaly. From the autopsy reports we gathered brain weight, fixation and staining methods, as well as details from the histological description.

Autopsy reports were assessed using a standardized semi-quantitative scoring system developed for this study to describe the degree of pathological involvement across anatomical regions. Information from the literature was classified on a scale from 0 (no involvement) to 3 (severe involvement), based on the terminology used by the original authors to describe the degree of pathology. Specifically, regions described as spared were assigned a score of 0; descriptions such as mild, minimal, or sparse involvement (and synonyms) were scored as 1; moderate involvement as 2; and severe or very severe involvement as 3.

Severity scoring was based on the overall description of pathological involvement within each region, including glycogen accumulation, gliosis, neuronal loss, and/or myelin loss. No weighting was applied to individual pathological features. In cases where only a single feature (e.g., glycogen accumulation) was described, this was used as the basis for classification. Reports that did not provide information on the degree of severity (e.g., only noting the presence of pathology without qualification) were excluded from severity scoring and from subsequent calculations.

An average severity score per anatomical region was calculated to enable a pictorial synopsis of the data. To reduce bias due to a limited number of observations, average scores were only calculated for regions described in autopsy reports of at least six subjects. The underlying extracted data and assigned severity scores are provided in Supplementary File 1.

Severity scoring was performed by a single reviewer (JD), and the extracted data and assigned scores were reviewed for consistency by a second author (JMPvdH). Formal inter-rater agreement was not assessed. Given the heterogeneity of reporting across studies, the scoring system should be regarded as a semi-quantitative tool to facilitate comparison between reports rather than a validated pathological scoring system.

## Results

### General

We identified 39 autopsy studies, comprising a total of 49 patients (Fig. 1) [9–47]. An overview of all included reports is shown in Table 1. Most reports were written before the advent of ERT in 1999 and describe patients who were not treated with ERT. Four studies consisting of seven autopsy reports were published after 1999 and describe autopsies on ERT treated patients [43, 44, 46, 47]. The median age of untreated patients was 6 months (range 1.2–17.0), while the median age of treated patients was 12 months (range 7.5–21.0).

**Table 1:** Overview of all included studies

First author (year)	Sex (F/M)	Age autopsy (mo)	Age first symptoms (mo)	Weight heart (g)	Weight brain (g)	CC	CWM	Di	BG	CB	BS	SC	Gen
Hertz (1936)	F	1.2	0	45	–			X					
Gunther (1939)	M	11.0	3	75	–						X	X	
Wachstein (1947)	F	2.3	0	99	–								X
Clement (1950)	F	6.0	0	110	–	X		X	X	X	X	X	
Di Sant’Agnese (1950)	F	4.0	0.5	90	–								X
Childs (1952)	M F	11.0 17.0	8 8	138 154	– –	X X		X		X X	X X	X X	
Selberg (1953)	M F	6.0 8.0	0 1.5	78 102	680 720	X X	X X	X X	X X	X X	X X	X X	
Schnabel (1958)	F	5.6	5	120	750	X	X	X	X	X	X	X	
Wilson (1960)	M	3.4	1	95	–		X					X	
Muller (1961)	M	8.5	1	170	–	X	X	X	X	X	X	X	
Stoeckle (1961)	M	6.7	1.5	120	753	X				X	X		
Caddell (1962)	F M	7.2 5.8	2 2.5	175 125	– –	X			X	X	X	X	X
Crome (1963)	F M	8.0 11.0	2.5 6	160 117	638 –	X X	X X	X X	X X	X X	X X	X X	
Gautier (1964)	F	7.9	5	–	–	X	X	X	X	X	X		
Kahana (1964)	F	5.4	3	60	–		X				X		
Lewis (1964)	M	3.5	2	212	–				X				
Loeb (1964)	M F	5.0 6.0	0 4	90 250	– –					X X	X X		
Dincsoy (1965)	M	3.5	3	150	520	X		X	X		X	X	
Mancall (1965)	M	8.0	0	200	820	X	X	X	X	X	X	X	
Toussaint (1965)	F	5.0	4	250	–					X			
Hernandez (1966)	F	1.2	0.5	145	–					X			
Joassin (1966)	M F	4.1 4.1	3 1	160 173	570 –	X X		X X	X X	X X	X X	X X	
Mekanik (1966)	F	9.0	4	110	–						X	X	
Hug (1967)	NR	6.0^c^	NR	–	–	X	X						
Garancis (1968)	F^b^	11.0	4	250	–					X			
Hogan (1969)	M	7.0	3	150	675	X	X	X		X	X	X	
Nihill (1970)	F	11.0	5.5	190	–					X			
Gambetti (1971)	NR	9.0	NR	–	–	X	X					X	
Leitriz (1973)	F M	13.0 4.0	3 1	– –	– –					X	X		X
Martin (1973)	M	6.0	1.5	–	–	X	X	X		X	X	X	
Sakurai (1974)	F	6.5	3	160	570								X
Hui (1985)	F	5.0	0	220	680	X		X	X	X	X	X	
Shotelersuk (2002)	F	9.0	0	195	720	X	X		X	X			
Teng (2004)	M	8.0	5	–	–	X					X	X	
Thurberg (2006)^a^	NR	14.0	NR	115	–	X				X		X	
Llerena (2008)^a^	NR NR	10.0 20.0	NR NR	– –	– –						X X	X X	
Dos Santos (2015)	M	5.0	2	168	742						X		
Pena (2015)^a^	F F M	21.0 12.0 7.5	2 3 5	– – –	1165 733 796	X	X X	X	X	X X	X X X	X X X	
Cerón-Rodríguez (2022)^a^	M	9.0	0.7	48	400				X		X	X	

*CC* cerebral cortex, *CWM* cerebral white matter, *Di* diencephalon, *BG* basal ganglia, *CB* cerebellum, *BS* brainstem, *SC* spinal cord, *Gen* only general description of central nervous system, *NR* not reported

^a^Treated with ERT

^b^Also reported by Hug (1967) as their case1

^c^Biopsy frontal cortex in alive patient

**Fig. 2:**
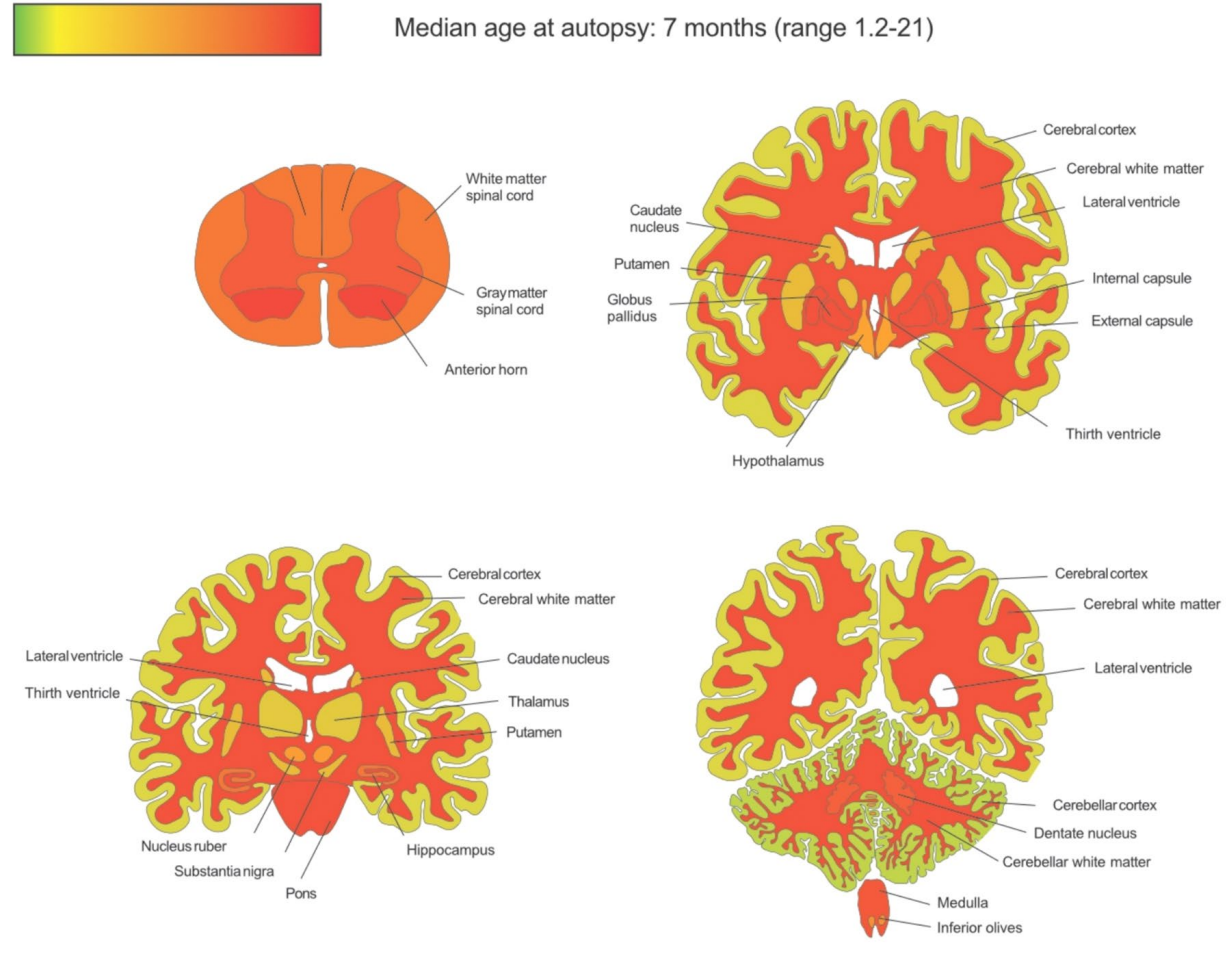
Anatomical representation of central nervous system involvement in classic infantile Pompe patients, with color coding reflecting histopathological findings identified by the systematic review. Colors range from green (no or minor involvement) to red (extensive involvement)

Although histologic abnormalities have been found throughout the central nervous system, there were clear differences in the degree of involvement, regarding both anatomical region and cell type (Table 1). We constructed an overview of the involvement of the most important anatomical structures using the scoring system (Fig. 2).

The cerebral and cerebellar white matter, globus pallidus, dentate nucleus of the cerebellum, motor nuclei of the brain stem, and anterior horn cells of the spinal cord were most frequently affected. These areas are discussed consecutively below.

### Cerebral cortex

The cerebral cortex was described in 25 cases [12, 14–16, 18–22, 26, 27, 30, 32, 34, 36, 38, 40–43, 46]. It was one of the least involved areas of the central nervous system. The cerebral cortex consists of six different layers. Four studies specifically describe these distinct cortical layers [21, 27, 34, 40], with marked glycogen accumulation and gliosis in layer I, which consists mainly of glial cells [21, 27, 40]. Most severe involvement of neurons was described by Mancall et al., in layers III–V [27] and by Hogan et al., in layers IV–VI [34]. No distinction was made between involvement of different cortical lobes.

Complete sparing of the cortex was found in 8 patients (median age 8 months, range 4.1–17.0) [14, 19, 22, 30, 41, 42]; mild or moderate glycogen accumulation, with variation between different cell types, was reported in 17 patients (median age 7 months, range 3.5–21.0) [12, 15, 16, 18, 20, 21, 26, 27, 32, 34, 36, 38, 40, 43, 46]. Affected neurons usually contained small quantities of glycogen [15, 16, 36, 38, 40, 43], although severe involvement with ballooning and destruction of neurons was described in two cases [27, 34]. Involvement of cortical glial cells varied considerably between studies. Glycogen accumulation in astrocytes varied from mild to severe [12, 16, 27, 34, 36, 38, 40], while oligodendroglia were mildly involved [16, 34, 36]. Moderate to severe gliosis of the cortex was described in two reports [27, 36].

### Cerebral white matter and lateral ventricles

Moderate to severe involvement of the cerebral white matter was found in the majority of case reports [15–17, 21, 22, 27, 32, 34, 36, 38, 41]. Normal myelination [21, 27, 38], as well as a decrease in myelin content or demyelination [15, 22, 23, 34], have both been reported. Although glycogen accumulation has been described in all types of glial cells, a considerable difference in severity of involvement was noted, and oligodendrocytes were relatively spared [22, 36, 38]. Glycogen accumulation was especially severe in astrocytes, with strong astrocytic proliferation and hypertrophy [17, 21–23, 27, 36, 38, 41, 46]. Several studies report moderate to intense fibrous gliosis throughout the white matter and accentuated around blood vessels [15, 16, 21–23, 27, 36, 38]. Glial glycogen storage and gliosis were also noted in the subependymal zone [27, 38, 40]. Moderate amounts of glycogen were seen in ependymal cells and vessel walls of the choroid plexus [14–16, 18, 21, 22, 30, 34, 38].

### Subcortical structures

Thalamic neuronal and glial glycogen storage was usually mild[9, 15, 16, 18, 22, 27, 30, 34, 38, 40, 46], with substantial variation between different thalamic nuclei [38], although no detailed information was available. Similar neuronal storage was reported in the hypothalamus [16, 27, 34, 38, 40]. Hippocampal involvement varied, with some reports noting differences between hippocampal regions; however, these differences were not consistently observed [16, 21, 38]. Hippocampal neurons stored mild to moderate amounts of glycogen; accumulation was mild in both astrocytes and oligodendroglia [12, 14–16, 20, 21, 26, 34, 38]. The putamen and caudate nucleus showed variable involvement, but usually not more than moderate glycogen deposition [15, 16, 18, 21, 22, 27, 30, 46]. In contrast with surrounding gray matter structures, extensive neuronal glycogen accumulation was consistently reported in the pallidum [15, 16, 18, 21, 22, 27, 30, 38].

### Cerebellum

The cerebellar cortex was spared or mildly involved [14–16, 18, 19, 21, 22, 25, 27, 30, 34, 41, 46]. While Purkinje cells were frequently spared [15, 16, 19–21, 25–27, 34, 35, 38, 43, 46] and glycogen accumulation was absent or sparse in the cortical granule cells, abundant accumulation was reported in Golgi type II cells [15, 16, 18, 21, 25, 27, 38]. Severe glycogen storage was consistently seen in the cerebellar white matter glia cells, resulting in astrocytic hypertrophy and gliosis [16, 21, 27, 34, 46]. With some exceptions, extensive neuronal glycogen accumulation was also found within the cerebellar nuclei, including the dentate nucleus [12, 14–16, 19–21, 25–28, 30, 34, 37, 38, 41, 46].

### Brainstem

Within the brainstem, severe glycogen storage has been found in all cranial nerve nuclei [10, 12, 14, 16, 18–23, 25–28, 30, 31, 34, 37, 38, 40, 42, 44–46]. The motor nuclei of the cranial nerve region were specifically described as one of the most severely affected areas of the central nervous system [14–16, 21, 22, 27, 30, 31, 34, 38]. Accumulation of glycogen in the pons and pontine nuclei varied from moderate to very severe [12, 14, 16, 18, 20–23, 25, 38, 45, 46]. Involvement of the substantia nigra and nucleus ruber was mild, and varied from complete sparing to moderate glycogen deposition [15, 16, 18, 21, 22, 30, 38, 46].

### Spinal cord

Glycogen storage was found throughout the spinal cord, with differences between cell types and anatomical regions. Neurons in the anterior horn were consistently described as most severely affected. Involvement comprised glycogen storage with distension and frequent destruction mostly of the large motor neurons [10, 12, 14–16, 18, 21, 27, 30, 31, 34, 36, 40, 43, 46]. Other neurons of the spinal cord were involved to a lesser degree, although glycogen storage has been reported throughout the gray matter [10, 12, 14–16, 21, 27, 31, 34, 36, 38, 40]. Glial cells were also affected, with storage in astrocytes, oligodendroglia, microglia and the ependymal cells of the central canal [15, 16, 20, 21, 36]. Gliosis has been reported in the anterior horn cells[38] and throughout the spinal cord [15, 16]. One study found myelin reduction[34].

### Central nervous system involvement in ERT treated patients

Seven autopsies of ERT-treated patients were described, of which 4 briefly [43, 44, 46, 47]. Differences between anatomical regions were rarely discussed in all of the reports, which makes it difficult to compare these results with pre-ERT autopsy studies. Overall the pattern of CNS involvement did not differ between treated and untreated patients. Glycogen was found in neurons of the cerebral cortex [43, 46] and glial cells, including astrocytes, of the cerebral white matter [46]. In the cerebellum, glycogen accumulation was found in neurons of dentate nucleus and glial cells of the white matter [46]. Purkinje cells were relatively spared [43, 46]. PAS positive material was also reported in basal ganglia [46, 47], thalamus [46] and brainstem [44, 46, 47]. In all cases glycogen storage was found in the spinal cord [43, 44, 46, 47] and anterior horn cells were specifically mentioned [43, 46]. In one case, cytoplasm vacuolar transformation in ganglionic neurons of the myenteric plexus of small intestine and colon was observed [47].

## Discussion

### General pathology

Central nervous system involvement has become increasingly apparent among long-term survivors with classic infantile Pompe disease and represents a relevant unmet medical need of this patient cohort. Working toward the development of innovative treatments which may address the CNS, we hereby performed a systematic review of published autopsy reports describing its involvement.

The available evidence is subject to limitations: most studies were conducted prior to the introduction of standardized histochemical and immunohistochemical techniques, resulting in substantial variability in tissue sampling, fixation, staining, analysis, and reporting. These methodological differences, together with small sample sizes, selective regional sampling, and the possibility of reporting bias, may influence the apparent distribution and severity of pathological findings and limit direct comparisons across studies and anatomical regions. Furthermore, because severity scores could only be assigned when explicit descriptions of severity were available and regions without sufficient information were excluded from scoring, incomplete reporting may have influenced the estimated distribution and severity of pathology across anatomical regions. Despite these limitations, the available studies provide valuable insight into the overall pattern of CNS involvement in classic infantile Pompe disease.

In summary, neuropathological studies showed that glycogen accumulation in classic infantile Pompe disease affects the CNS in a region- and cell-specific manner. The cerebral cortex was relatively spared, whereas cerebral white matter, particularly astrocytes, showed severe involvement with perivascular gliosis. Subcortical gray matter, especially the pallidum, was variably affected, while the cerebellar dentate nucleus, brainstem motor nuclei, and spinal anterior horn neurons consistently demonstrate marked glycogen storage and gliosis. Importantly, available autopsy reports of ERT-treated infants are limited in number, detail, and age at death (median age of death 12 months, range 7.5–21.0). Nevertheless, despite the relatively young age of these patients, CNS pathology was still documented across cortical, subcortical, cerebellar, brainstem, and spinal regions. These observations are biologically plausible given that intravenously administered ERT has been shown to have no meaningful penetration of the blood–brain barrier and is therefore unable to correct the enzyme deficiency within the CNS [48, 49]. However, the currently available post-mortem evidence remains sparse.

### Relationship between histopathological, radiological, biochemical, and clinical findings

Before investigating the relationship between the histopathological and clinical findings, it is important to consider the wide gap between the young age at autopsy (12 months for the treated patients) and the age currently reached by a few classic infantile patients (around 25 years old). Additionally, CNS involvement progresses over time, with some patients showing a faster rate of progression, while others deteriorate more slowly.

Autopsy studies consistently identified the cerebral white matter as extensively involved in young patients with classic infantile Pompe disease. Such histopathological findings have both a well-known radiological, biochemical, and clinical correlation. Interestingly, however, there appears to be a marked temporal discrepancy between pathological and clinical evidence: despite severe white matter involvement at autopsy at a young age, radiological abnormalities become first evident only from approximately 2–3 years of age, followed by biochemical changes from around 5 years, and clinical manifestations generally emerging from around 8 to 10 years of age.

Radiologically, corresponding MRI abnormalities range from delayed myelination in infancy to progressive white matter involvement thereafter [2, 50–54], with the earliest detectable changes reported around 2–3 years of age [2, 51]. Biochemically, a longitudinal increase of serum neurofilament light chain (NfL) [51, 55], an accepted biomarker of neuroaxonal and astroglia damage, and plasma glial fibrillary acidic protein (GFAP) [56], indicating astrocytic dysfunction, have been reported in classic infantile Pompe disease from approximately 5 years of age onward. Clinically, white matter abnormalities correlate with slower processing speed and progressive cognitive decline, which may become evident on neuropsychological testing around the age of 8–10 years old [5, 50–52, 57, 58]. This temporal gap between early pathological changes and later radiological, biochemical, and clinical expression suggests that underlying disease processes likely evolve gradually over time, with alterations accumulating until a threshold is reached at which they become detectable by current imaging modalities or clinical assessment. However, the precise nature of these processes remains insufficiently understood. Further autopsy studies in long-term survivors are needed to better elucidate the pathological correlates of disease progression and strengthen clinicopathological correlations.

We previously hypothesized that cognitive decline in classic infantile Pompe disease could be understood within the Parieto-Frontal Integration Theory (P-FIT), which proposes that cognitive functioning depends on efficient communication between brain regions, mediated primarily by intact white matter tracts such as the superior longitudinal fasciculus. Supporting this hypothesis, diffusion tensor imaging (DTI) in a classic infantile Pompe cohort revealed abnormalities in this tract, indicating structural disruption [59]. Consistent with diffuse white matter dysfunction and delayed neuronal signal transmission, slowing of background electrical activity on electroencephalography has also been observed in a cohort of classic infantile Pompe patients who developed epilepsy [60], even though further studies are required to investigate such a hypothesis.

Beyond cognitive decline, widespread damage to white matter, including the corticospinal tract, is likely to clinically manifest with upper motor neuron signs (UMN), such as spasticity and hyperreflexia. Although UMN signs were traditionally not observed in classic infantile Pompe patients, potentially because they were masked by severe muscle weakness, reports on patients with UMN signs are growing [5, 61]. While UMN signs have been only recently recognized, it is well known that patients with classic infantile Pompe disease often develop distal muscle weakness [3].

The underlying mechanisms of distal muscle weakness are not fully understood and appear to differ between patients. Evidence points to both myopathic and neurogenic contributions. In one well-documented case, rapid distal weakness under ERT was associated with severe myopathy but only mild peripheral nerve involvement [62]. Conversely, a series of patients who developed bilateral foot drop under ERT showed electrophysiological and histological evidence of coexisting motor neuronopathy, hypothesized to result from glycogen accumulation in spinal motor neurons [63]. This interpretation aligns with autopsy studies demonstrating extensive glycogen deposition in anterior horn cells, although the extent to which this contributes to clinically overt lower motor neuron (LMN) signs remains uncertain. LMN involvement is classically characterized by flaccid weakness, muscle atrophy, and hyporeflexia; however, these neurogenic features may be clinically subtle or obscured in the presence of severe myopathy. Thus, distal weakness in Pompe disease likely reflects a complex interplay between progressive myopathy and, in some patients, coexisting neurogenic involvement. A neurogenic component cannot be excluded also for bulbar muscle weakness and facial myopathy, commonly observed in classic infantile Pompe patients [6, 64], which are partly due to incomplete glycogen clearance in muscle despite ERT.

In contrast to the white matter and motor neuron involvement highlighted in this systematic review, histopathological studies in young patients indicate that cortical neurons are affected with a higher degree of variability and severity. While autopsies show less gray matter involvement in younger children, the pathology is likely evolving, as reflected by the progressive clinical presentation. In imaging studies, hyperintensities of subcortical structures including basal ganglia and thalamus are detected around the age of 8 years and 9.5 years, respectively [2]. Conversely, while motor nuclei of the brainstem generally showed extensive involvement in autopsy studies, MRI of the brainstem generally shows no abnormalities until a later age [2, 52]. Atrophy, which can be gray and/or white matter related, as well, is mostly observed at an older age in a subset of patients. CNS autopsy studies in older patients would contribute to understanding the extent of gray matter involvement. As mentioned previously, clinical manifestations of subcortical gray matter and cortical structures damage, such as hypertonia, clonus, spasticity, hemiplegia and cerebellar signs, are increasingly being reported in older patients. Moreover, twenty-three cases of patients with classic infantile Pompe disease who developed epilepsy have been published to date, typically presenting after the age of 7 ([5, 60, 65]). Both cognitive decline and epilepsy may be secondary to both white matter and gray matter pathology.

Other CNS manifestations in classic infantile Pompe disease include hearing and vision impairments. Although autopsy studies of the ear in patients are lacking, Pompe mouse models showed glycogen storage in cochlear structures and spiral ganglia, likely contributing to the sensorineural hearing loss frequently reported in patients [4, 6, 66–69]. In line with known histopathology, mildly prolonged interpeak latencies on auditory brainstem response (ABR) suggest subclinical brainstem involvement in some cases [69]. Ocular involvement has also been reported, with glycogen deposition in multiple ocular tissues including the retina, as well as in ocular muscles, where it contributes to ptosis and extraocular motility disorders [70–74]. Clinically, myopia, ptosis, and ocular movement abnormalities have been observed, but blindness, whether originating from retinal or visual tract involvement, has not been reported to date. Nevertheless, careful long-term follow-up of visual function remains important, as such complications may emerge as the cohort of treated patients becomes larger and older. A single case of intermediate uveitis with retinal detachment in a patient might further suggest that additional ocular complications may emerge with longer survival on ERT [75].

### Hypothesized pathophysiological mechanisms of central nervous system involvement in classic infantile Pompe disease

Autopsy studies consistently identify astrocytes as the most prominently affected cell type within the central nervous system in classic infantile Pompe disease. This vulnerability likely reflects their distinctive metabolic role: glycogen in the brain is almost exclusively localized within these cells, where it normally serves as a rapidly mobilizable reserve of energy. Although these histopathological observations are well supported, the causal links between glycogen accumulation and downstream neuronal dysfunction remain incompletely understood. The mechanistic interpretations discussed below are primarily inferred from established neurobiology, biomarker studies in patients, and experimental findings in animal models.

Recent advances in tissue fixation and analytical techniques have improved our understanding of brain glycogen distribution and function [76, 77]. Physiologically, glycogen is heterogeneously distributed throughout the brain, being most abundant in astrocytes and present to a lesser extent in neurons and microglia. Astrocytic glycogen plays a central role in supporting neuronal activity, including through the astrocyte–neuron lactate shuttle and the glutamate–glutamine cycle, thereby sustaining axonal conduction, synaptic transmission, and memory consolidation [76, 77].

Human autopsy studies and knockout mouse models both demonstrate extensive astrocytic glycogen accumulation, astrocytic hypertrophy, and microglial activation, suggesting that gliosis may represent an early and central component of the white matter disease process [7, 78–80]. In classic infantile Pompe patients, increased blood levels of GFAP and NfL provide indirect evidence of astrocytic activation and axonal injury, respectively. Together, these biomarkers may provide in vivo findings that are broadly consistent with the underlying neuropathology and animal studies.

**Fig. 3:**
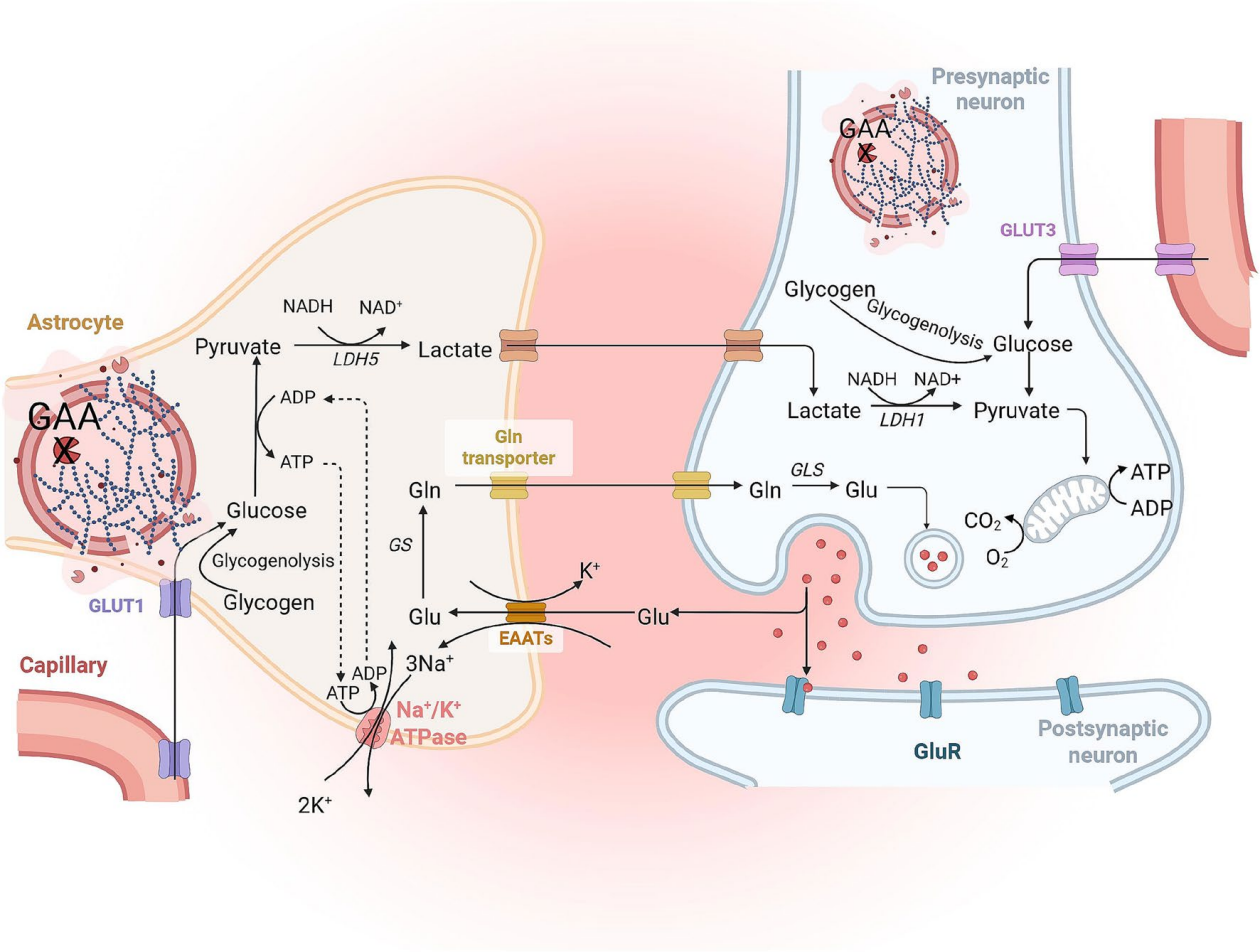
Hypothesized pathogenesis of central nervous system (CNS) involvement in classic infantile Pompe disease. Autopsy studies and alpha-glucosidase (GAA) deficient mouse models identify astrocytes as the most affected cell type, characterized by glycogen accumulation, hypertrophy, and associated microglial activation [7, 78, 79]. Although astrocytes exhibit the most consistently reported and severe pathology, neurons and oligodendrocytes may also be involved, either through direct lysosomal glycogen storage or secondary injury resulting from astrocytic dysfunction. Based on general neurobiological principles and animal studies, we hypothesize that astrocyte impairment may disrupt critical astrocyte-neuron interactions. Such interactions include the astrocyte–neuron lactate shuttle, in which astrocytic glycolysis generates lactate from pyruvate via lactate dehydrogenase 5 (LDH5), and lactate is exported to neurons where lactate dehydrogenase 1 (LDH1) converts it back to pyruvate to support mitochondrial oxidative phosphorylation and ATP synthesis. Glucose uptake into astrocytes and neurons occurs via glucose transporters 1 and 3 (GLUT1 and GLUT3, respectively), with astrocytic glycogen serving as a local energy reserve. In parallel, dysfunction of the glutamate–glutamine cycle may occur, whereby uptake of glutamate (Glu) by astrocytes from the synapse, via excitatory amino acid transporters (EAATs), followed by conversion of glutamate to glutamine (Gln) by glutamine synthetase (GS), and subsequent neuronal reconversion of glutamine to glutamate by glutaminase (GLS), is compromised. Postsynaptic glutamate signaling is mediated by glutamate receptors (GluR). These metabolic and neurotransmitter recycling processes are tightly coupled to astrocytic regulation of extracellular ion homeostasis, including Na^+^, K⁺, and Ca^2^⁺ fluxes, as well as redox balance (NAD⁺/NADH). Disruption of these integrated pathways is hypothesized to contribute to neuronal dysfunction, impaired myelination, and progressive CNS involvement in classic infantile Pompe disease. These mechanisms remain largely inferential and have not been directly demonstrated in human disease. Created with BioRender.com

Although astrocytes show the most prominent glycogen storage in young patients, involvement of neurons and oligodendrocytes has also been demonstrated. These cell types may be directly affected by glycogen accumulation within their lysosomes; additionally, neurons and oligodendrocytes may be secondarily injured by astrocytic dysfunction, resulting in impaired metabolic support, defective neurotransmitter clearance, imbalances in the extracellular ionic milieu (including calcium, potassium, and sodium) and dysregulation of myelination processes [81–83]. These proposed mechanisms are summarized schematically in Fig. 3. Oligodendrocyte involvement appears less evident in young patients and only mild pathology was described in autoptic reports. However, this limited early involvement should be interpreted in the context of normal myelination patterns.

The typical progressive brain MRI pattern consists of symmetrical white matter abnormalities with relative sparing of U-fibers, a pattern that resembles that observed in several leukodystrophies, including metachromatic leukodystrophy. While this observation may be compatible with oligodendrocyte involvement, it does not establish oligodendrocyte dysfunction as the primary pathological process, particularly given the prominent astrocytic pathology observed in autopsy studies. Interestingly, a periventricular rim has also been observed in Pompe disease, a radiological feature that is characteristically associated with Alexander disease, an astrocytopathy. However, radiological overlap does not necessarily imply shared cellular mechanisms, and the significance of these observations remains unknown. Importantly, relative sparing of U-fibers may reflect the normal sequence of myelination and therefore does not exclude oligodendrocyte involvement. Their late myelination, higher metabolic demands, and less compact structure may render these fibers relatively resistant in the early stages of disease [84–87]. Thus, the preserved U-fibers in young Pompe patients may reflect developmental timing rather than true protection, with oligodendrocyte involvement hypothesized to become more prominent as the disease advances, consistent with patterns observed in other leukodystrophies and in older individuals.

### Limitations

Several limitations should be considered when interpreting the available neuropathological data. First, a number of studies provide insufficient detail regarding the tissues examined and the procedures used for tissue preparation, while others applied different histological methods, complicating direct comparison across studies. Second, the examined CNS regions were not consistent between reports, with most studies focusing on a limited set of areas. Finally, the total number of autopsy cases remains small, and most patients were very young at the time of death, including those treated with ERT, which restricts the ability to assess age-related progression of pathology. Taken together, these factors highlight the need for cautious interpretation of cross-study comparisons. Despite these limitations, however, the histological findings reported to date are relatively consistent across anatomical regions and cell types, supporting the robustness of the observed patterns.

## Conclusion

CNS involvement in classic infantile Pompe disease is increasingly recognized as widespread and clinically relevant, particularly as current enzyme replacement therapy does not adequately address CNS disease. The precise cascade leading to CNS pathology remains to be clarified, but it is evident that cerebral white matter is particularly vulnerable to disrupted lysosomal glycogen metabolism in young patients; the extension of gray matter disease and its translation into symptoms is not yet defined, although clinical descriptions in line with upper motor neuron involvement are increasingly growing. Evidence of distal muscle weakness, sensorineural hearing loss, and ocular manifestations further illustrates that glycogen storage affects multiple components of the nervous system.

These observations underscore the need for systematic, longitudinal monitoring of affected patients, including neurological examination, cognitive assessment, brain MRI, and the development of reliable biomarkers of CNS disease. Future therapies will need to target not only skeletal muscle but also the central and peripheral nervous system. A key unresolved question is whether CNS pathology is reversible; encouragingly, astrogliosis has been shown to reverse in Pompe knock-out mice treated with lentiviral modified human stem cell transplantation [7], offering hope for future interventions.

## Supplementary Information

Below is the link to the electronic supplementary material.

Supplementary file1 (DOCX 16 KB)

Supplementary file2 (XLSX 72 KB)

## Data Availability

All data supporting the findings of this study are available within the paper and its Supplementary Information.
