# Supplementary material for: Central nervous system histopathological findings in classic infantile Pompe disease: a systematic review with clinical relevance
Source: J Neurol. 2026 Jul 31;273(8):496. doi: 10.1007/s00415-026-13991-y (PMC13427966; doi:10.1007/s00415-026-13991-y)
Supplement: Supplementary file 1 — Supplementary file1 (DOCX 16 KB) [file 415_2026_13991_MOESM1_ESM.docx]

**Appendix A.**

**Systematic search performed on 16-12-2025.**

**Embase.com**

('glycogen storage disease type 2'/exp OR ('enzyme deficiency'/de AND 'glucan 1,4 alpha glucosidase'/de) OR ('glucosidase'/exp AND ( 'lysosome storage disease'/de OR 'glycogen storage disease'/de)) OR ((glycogen* NEAR/3 storage NEAR/6 (2 OR ii OR generali*)) OR pompe OR ((diffuse OR 2 OR ii) NEAR/3 glycogenos*) OR ((acid-maltase OR glucosidase OR GAA ) NEAR/3 deficien*) OR (glycogen* NEAR/3 storage AND (type-2 OR type-ii ))):ab,ti) AND ('autopsy'/exp OR 'pathology'/de OR (autops* OR post-mort* OR postmort* OR patholog*):ab,ti)

('glycogen storage disease'/exp OR ((glycogen* NEAR/6 (generali* OR storage OR disease*))):ab,ti) AND [1800-1975]/py

**Medline Ovid**

("Glycogen Storage Disease Type II"/ OR "Glucan 1,4-alpha-Glucosidase"/df OR (exp "glucosidases"/ AND ( "Glycogen Storage Disease"/)) OR ((glycogen* ADJ3 storage ADJ6 (2 OR ii OR generali*)) OR pompe OR ((diffuse OR 2 OR ii) ADJ3 glycogenos*) OR ((acid-maltase OR glucosidase OR GAA ) ADJ3 deficien*) OR (glycogen* ADJ3 storage AND (type-2 OR type-ii ))).ab,ti.) AND (autopsy/ OR exp pathology/ OR (autops* OR post-mort* OR postmort* OR patholog*).ab,ti.)

(exp Glycogen Storage Disease/ OR ((glycogen* ADJ6 (generali* OR storage OR disease*)) OR pompe).ab,ti.)

Limit 1 to yr=1800-1966

**Web of science**

TS=((((glycogen* NEAR/2 storage NEAR/5 ("2" OR ii OR generali*)) OR pompe OR ((diffuse OR "2" OR ii) NEAR/2 glycogenos*) OR ((acid-maltase OR glucosidase OR GAA ) NEAR/2 deficien*) OR (glycogen* NEAR/2 storage AND (type-2 OR type-ii)))) AND ((autops* OR post-mort* OR postmort* OR patholog*)))

**Cochrane CENTRAL**

(((glycogen* NEAR/3 storage NEAR/6 (2 OR ii OR generali*)) OR pompe OR ((diffuse OR 2 OR ii) NEAR/3 glycogenos*) OR ((acid next maltase OR glucosidase OR GAA ) NEAR/3 deficien*) OR (glycogen* NEAR/3 storage AND (type next 2 OR type next ii))):ab,ti) AND ((autops* OR post next mort* OR postmort* OR patholog*):ab,ti)

**Overview of identified publications**

| Year | Total | Remove duplicates |
| --- | --- | --- |
| Embase.com | 2313 | 1380 |
| Medline Ovid all | 987 | 980 |
| Web of science | 428 | 85 |
| Cochrane CENTRAL | 6 | 3 |
| **Total** | **3734** | **2448** |
